# Blockade of lymphotoxin-beta receptor signaling reduces aspects of Sjögren's syndrome in salivary glands of non-obese diabetic mice

**DOI:** 10.1186/ar2617

**Published:** 2009-02-18

**Authors:** Margaret K Gatumu, Kathrine Skarstein, Adrian Papandile, Jeffrey L Browning, Roy A Fava, Anne Isine Bolstad

**Affiliations:** 1 Section for Pathology, The Gade Institute, University of Bergen, Haukeland University Hospital, Jonas Lies vei 65, N-5021 Bergen, Norway; 2 Department of Immunobiology, Biogen Idec, 12 Cambridge Center, Cambridge, MA 02142, USA; 3 Department of Veterans Affairs Medical Center, 215 North Main Street, White River Junction, VT 05009, USA; 4 Department of Micro/Immunology, Dartmouth Medical School, 1 Rope Ferry Road, Hanover, NH 03756, USA; 5 Department of Clinical Dentistry – Periodontics, University of Bergen, Aarstadveien 17, N-5009 Bergen, Norway

## Abstract

**Introduction:**

The lymphotoxin-beta receptor (LTβR) pathway is important in the development and maintenance of lymphoid structures. Blocking this pathway has proven beneficial in murine models of autoimmune diseases such as diabetes and rheumatoid arthritis. The aim of this study was to determine the effects of LTβR pathway blockade on Sjögren syndrome (SS)-like salivary gland disease in non-obese diabetic (NOD) mice.

**Methods:**

The course of SS-like disease was followed in NOD mice that were given lymphotoxin-beta receptor-immunoglobulin fusion protein (LTβR-Ig) starting at 9 weeks of age. Treatment was given as a single weekly dose for 3, 7, or 10 weeks. Age-matched NOD mice treated with mouse monoclonal IgG1, or not treated at all, were used as controls. The severity of inflammation, cellular composition, and lymphoid neogenesis in the submandibular glands were determined by immunohistochemistry. Mandibular lymph nodes were also studied. Saliva flow rates were measured, and saliva was analyzed by a multiplex cytokine assay. The salivary glands were analyzed for CXCL13, CCL19, and CCL21 gene expression by quantitative polymerase chain reaction.

**Results:**

Treatment with LTβR-Ig prevented the increase in size and number of focal infiltrates normally observed in this SS-like disease. Compared with the controls, the submandibular glands of LTβR-Ig-treated mice had fewer and smaller T- and B-cell zones and fewer high endothelial venules per given salivary gland area. Follicular dendritic cell networks were lost in LTβR-Ig-treated mice. CCL19 expression was also dramatically inhibited in the salivary gland infiltrates. Draining lymph nodes showed more gradual changes after LTβR-Ig treatment. Saliva flow was partially restored in mice treated with 10 LTβR-Ig weekly injections, and the saliva cytokine profile of these mice resembled that of mice in the pre-disease state.

**Conclusions:**

Our findings show that blocking the LTβR pathway results in ablation of the lymphoid organization in the NOD salivary glands and thus an improvement in salivary gland function.

## Introduction

Sjögren syndrome (SS) is a chronic autoimmune disorder characterized by lymphocytic infiltrates of the exocrine glands. Patients present clinical symptoms of dry eyes and dry mouth. The exocrinopathy may occur alone (primary SS) or in association with another autoimmune disorder (secondary SS) such as rheumatoid arthritis or systemic lupus erythematosus [1]. Inflammatory reactions in the lacrimal and salivary glands with lymphoid neogenesis, and specifically germinal center formation, are reported in SS patients [2-5].

The lymphotoxins (LTα and LTβ) and LIGHT (a cytokine homologous to LTs that shows inducible expression and competes with herpes simplex virus glycoprotein D for herpes virus mediator [HVEM], a receptor induced on T cells) and their receptors are part of the tumor necrosis factor (TNF) superfamily. LTα exists in both secreted and membrane-bound forms. The membrane-bound form is a complex of LTα and LTβ [6], forming in humans a predominant LTα_1_β_2 _heteromer [7]. This ligand binds the LTβ receptor (LTβR) [8] along with LIGHT [9]. For a detailed review of LT and LIGHT signaling, see [10].

The LTβR signaling pathway is important in the development of organized lymphoid structures [10-18]. In the differentiated tissue, the LTβR signaling pathway is involved in the control of expression of chemokines and adhesion molecules that aid in the movement of lymphocytes and in their compartmentalization into T- and B-cell zones, high endothelial venule (HEV) development, and follicular dendritic cell (FDC) network formation and in the positioning and numbers of dendritic cells [19]. This signaling pathway is also crucial in lymphoid neogenesis at sites of inflammation [20]. Lymphoid neogenesis is reported in human chronic inflammatory diseases (including SS) and associated mouse models (reviewed in [21,22]).

The efficacy of LTβR pathway blockade (that is, the blocking of activation of LTβR by either of its ligands via the pharmacological inhibitor LTβR-immunoglobulin fusion protein [LTβR-Ig]) has been studied in many murine disease models (reviewed in [19]), and a human version was used in rheumatoid arthritis clinical trials [13].

In non-obese diabetic (NOD) mice, treatment with LTβR-Ig prevented insulin-dependent diabetes mellitus (IDDM) and reversed insulitis [23,24]. In addition, soluble LTβR-Ig transgene expression on the NOD background blocked diabetes development [25]. In addition to spontaneously developing IDDM, the NOD mouse spontaneously develops lymphocytic infiltrates in the exocrine glands, with associated glandular dysfunction [26]. Although the *Idd3 *and *Idd5 *susceptibility loci for diabetes have a role in the development of sialadenitis, the IDDM and sialadenitis are two independent autoimmune events [27-29].

The aim of the present study was to analyze the effects of LT/LIGHT axis blockade by the pharmacological inhibitor LTβR-Ig on SS-like salivary gland disease in female NOD mice, introducing the inhibitor prior to the development of the SS-like disease. The study confirmed that blocking the LTβR pathway results in ablation of the lymphoid organization in the NOD salivary glands, leading to an improvement in salivary gland function.

## Materials and methods

### Animals and treatment protocols

**Figure 1:**
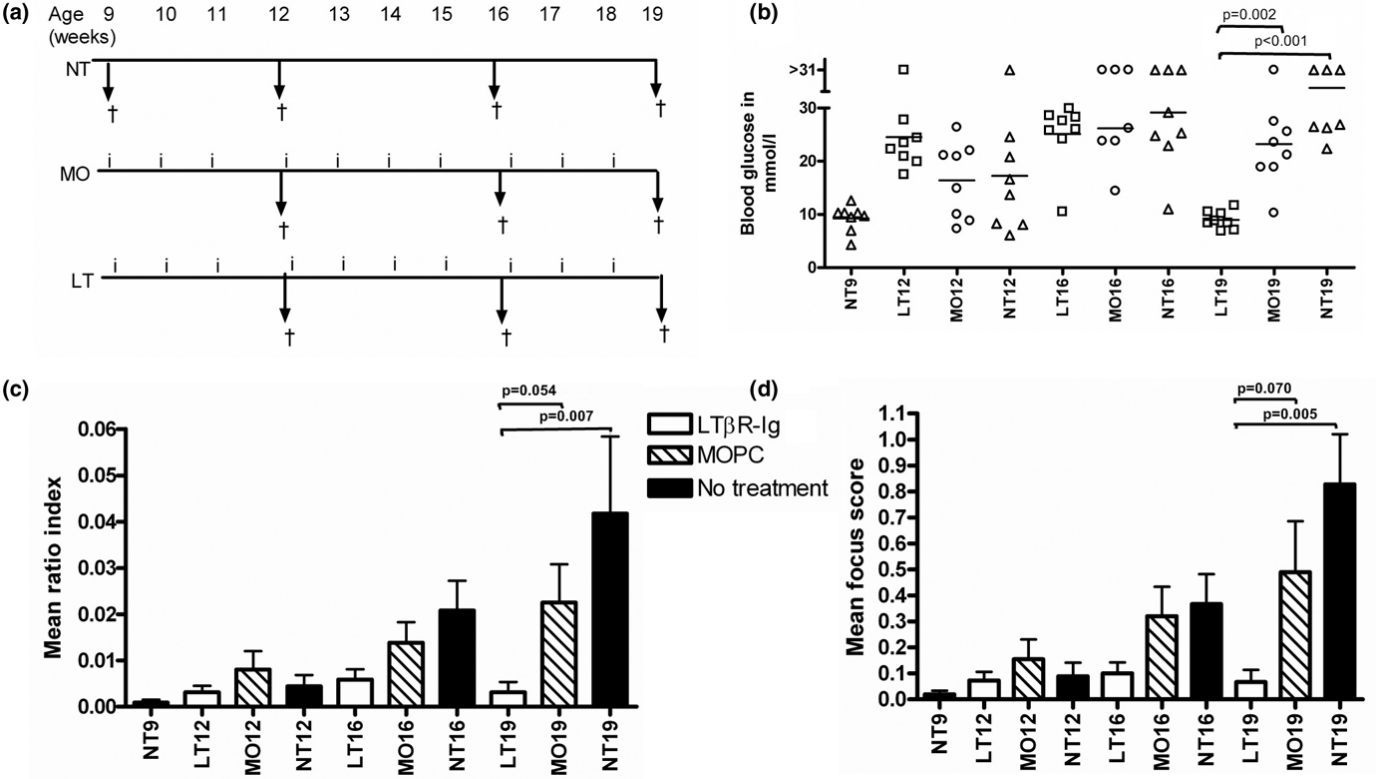
Experimental protocol and the effect of lymphotoxin-beta receptor-immunoglobulin fusion protein (LTβR-Ig) treatment on blood glucose levels and on submandibular gland inflammation. **(a) **A weekly injection (i) of either LTβR-Ig (LT) or mouse monoclonal IgG1 (MOPC 21) (MO). An isotype-matched control Ig was administered. Age-matched mice that were not treated (NT) were also examined. Saliva flow rate measurements were performed, and the mice were then euthanized and salivary glands were harvested (†). **(b) **Blood glucose levels of individual mice in the experiment. Horizontal lines indicate the median. At 19 weeks, the LTβR-Ig-treated group blood glucose level was significantly lower than that of the age-matched untreated mice (*P *< 0.001). Symbols refer to individual mice treated with LTβR-Ig (□), MOPC 21 (○), or no treatment (Δ). Each symbol indicates data from one mouse, and data were obtained on the day of euthanasia. The ratio index **(c) **and focus score **(d) **were used to analyze the level of inflammation in the submandibular glands. Each group comprised seven or eight mice. Bars indicate the mean and the standard error of the mean.

Female NOD/LtJ mice were purchased from The Jackson Laboratory (Bar Harbor, ME, USA) and maintained in accordance with standard animal housing and welfare guidelines. The protocol is shown in Figure 1a. In summary, eight mice that were not treated (NT) were euthanized at 9 weeks (NT9 group) and formed the baseline of the experiment. Beginning at 9 weeks of age, groups of eight mice were given weekly intraperitoneal injections of 100 μg of either LTβR-Ig (murine receptor fused to mouse IgG1, which has been described previously [30]) or mouse monoclonal IgG1 (MOPC 21) for 3, 7, and 10 weeks. Mice were then euthanized 1 week after the last injection (that is, at 12, 16, or 19 weeks). The LTβR-Ig-treated mice are subsequently referred to as LT12, LT16, and LT19 and the MOPC 21-treated ones as MO12, MO16, and MO19, respectively. Groups of age-matched control mice that were NT (with either LTβR-Ig or MOPC 21) were also investigated and are subsequently referred to as NT12, NT16, and NT19. The experimental protocol (research number 2006012BB) was approved by the National Animal Research Authority of Norway.

### Diabetes monitoring

The mice were monitored weekly for changes in weight. On the day of euthanasia, blood glucose was measured after 2 hours of fasting (with water *ad libitum*) using the HemoCue Glucose 201 RT test kit (HemoCue Norge, Oslo, Norway).

### Salivary gland function analysis

On the day of euthanasia, a subcutaneous injection of a combination of ketamine and medetomidine hydrochloride (0.15 mL per 20 g of body weight) was used to anaesthetize the mice. Saliva secretion was stimulated by intraperitoneal pilocarpine in saline (0.1 mL per 20 g of body weight), collected for 10 minutes by a pipette, and transferred to pre-weighed microtubes. The volume of the saliva sample was determined and standardized against the weight of the individual mouse. The saliva samples were then frozen at -80°C.

### Multiplex cytokine analysis of saliva

Saliva samples were analyzed for cytokines using a mouse cytokine twenty-plex kit (catalog number LMC006, BioSource; Invitrogen Corporation, Carlsbad, CA, USA). The instructions of the manufacturer were adhered to. The assay was measured by a Luminex 100™ instrument (Luminex Corporation, Austin, TX, USA) and analyzed using StarStation 2.0 software (Applied Cytometry Systems, Sacramento, CA, USA).

### Salivary gland inflammation analysis

Submandibular and sublingual salivary glands were excised and either snap-frozen in isopentane by liquid nitrogen and stored at -80°C or placed in 4% formalin, left overnight, and processed under standard techniques to obtain paraffin blocks. The submandibular glands of all of the mice were examined histologically after hematoxylin-and-eosin staining of two to six sections at different levels in the gland and morphometrically analyzed using a Leica DMLB light microscope (Leica, Wetzlar, Germany) connected to an Olympus Color View III camera (Olympus, Tokyo, Japan) and AnalySIS software (Soft Imaging System, Münster, Germany). The focus score [31] (number of foci made up of at least 50 mononuclear cells per square millimeter of glandular tissue) and ratio index [32] (ratio of the area of inflammation to the total area of the gland) were determined.

### Antibodies

The antibodies used were anti-B220, rat IgG2B, clone RA3-6B2 (R&D Systems, Minneapolis, MN, USA) (stains B cells); anti-CD4, rat IgG2B, clone YTS191.1 (Dako Denmark A/S, Glostrup, Denmark) (stains T cells); anti-CD21/CD35, clone 7G6 (BD Pharmingen, San Diego, CA, USA) (stains FDCs); anti-peripheral (lymph) node addressin (anti-PNAd) carbohydrated epitope, clone MECA-79 (BD Pharmingen) (stains HEVs); anti-CXCL13 goat IgG, lot numbers COZ025081 and COZ026061 (R&D Systems); and anti-CCL19 goat IgG, lot number BUL026021 (R&D Systems). The avidin-biotin complex technique was used for the immunohistochemistry analysis as described previously [32]. Frozen and paraffin sections of salivary glands and mandibular lymph nodes (processed in same manner as indicated above for salivary glands) were analyzed.

### Analysis of T- and B-cell zones and high endothelial venules

The submandibular glands were examined histologically by authors MKG and KS in a blinded fashion after immunohistochemical single-staining with CD4 (stains T cells), B220 (stains B cells), and PNAd (stains HEVs) and morphometrically analyzed as stated above. The ratio of the zone of T-cell aggregates or B-cell aggregates to the total area of the submandibular gland was determined. Isolated stained cells were not included in this analysis. The number of HEVs per given submandibular gland area was also analyzed.

### Quantitative polymerase chain reaction

Dissected submandibular glands from seven or eight mice were placed in RNA *later*^® ^(Ambion, Inc., Austin, TX, USA) at 4°C overnight before being frozen at -80°C until use. Organs were disrupted in TRIzol^® ^Reagent (Invitrogen Corporation). RNA was extracted using RNeasy (Qiagen Inc., Valencia, CA, USA). RNA was DNase-treated and cDNA was prepared using a cDNA archive kit (Applied Biosystems, Foster City, CA, USA). Quantitative polymerase chain reaction (QPCR) was performed as described previously [33]. All RNA samples were run in quadruplicate. The data were normalized to glyceraldehyde 3-phosphate dehydrogenase (GAPDH) as an endogenous control. QPCR primers and probes were as follows: CXCL13: forward GTAAAACGCAGGCTTCCAAAA, reverse GATGGCATTGCACCAGCTT, and FAM probe AGTCTCCAGAAGGTTC; CCL19: forward CCTCGGCCTCTCAGATTCTTG, reverse GGCAGGCCACAGAGAGTGA, and FAM probe CACACAGTCTCTCAGGCT; and CCL21: forward GGGCTGCAAGAGAACTGAACA, reverse GGCGGGCTACTGGGCTAT, and FAM probe ACACAGCCCTCAAGAG.

### Serum analysis for LTβR-Ig levels by enzyme-linked immunosorbent assay

A 96-well plate was coated with hamster anti-murine LTβR ACH6 (Biogen Idec, Cambridge, MA, USA) and incubated overnight. The plate was blocked with 1% bovine serum albumin (BSA) in phosphate-buffered saline (PBS). Sera, at dilution 1:900 in 1% BSA in PBS, were added to the wells and incubated for 1 hour. Peroxidase-conjugated donkey anti-mouse IgG (code number 715-036-150; Jackson ImmunoResearch Laboratories, Inc., West Grove, PA, USA) was then incubated for 35 minutes. Samples were run in duplicate. Serial dilutions of LTβR-Ig were used to construct the standard curve. Plates were washed with PBS (pH 7.0) with 0.05% Tween. Absorbance values were measured by a Multiskan^® ^microplate photometer (Labsystems Inc., Franklin, MA, USA) and analyzed by Ascent software (Thermo Fisher Scientific Inc., Waltham, MA, USA).

### Statistics

Parametric data were analyzed using one-way between-groups analysis of variance (ANOVA) with Tukey HSD (honestly significant difference) *post hoc *test and the Student *t *test. Two-way between-groups ANOVA was undertaken for specific comparisons of LTβR-Ig and MOPC 21 treatment on blood glucose levels, inflammation, and saliva flow rates. The Mann-Whitney *U *test was used for non-parametric data. Pearson's correlation was used to describe linear relationships. Saliva cytokines were Z-standardized. SPSS version 15.0 (SPSS Inc., Chicago, IL, USA) was used for these analyses. The Kruskal-Wallis test and Dunn's multiple comparison test were used for the LTβR-Ig level analysis using GraphPad version 4 (GraphPad Software, Inc., San Diego, CA, USA). *P *values of less than 0.05 were considered significant.

## Results

### Effect of LTβR-Ig treatment on blood glucose levels

As highlighted in the Introduction, several studies have reported successful prevention of diabetes development in NOD mice upon treatment with LTβR-Ig. We used the blood glucose levels as an in-house control to determine the efficacy of LTβR-Ig treatment. Both the number of injections and the type of treatment (either LTβR-Ig or MOPC 21) played a role in determining the glucose levels, as shown by the significant interaction effect (*P *< 0.001). This finding was underscored by the low glucose levels at 16 weeks in the LT16 group compared with age-matched controls, and at 19 weeks, the LT19 group glucose levels were not only lower than those of the LT16 group, but also significantly different from those of age-matched controls (Figure 1b).

### LTβR-Ig treatment inhibits submandibular gland inflammation

Submandibular salivary glands were examined for inflammation. The ratio index and focus score were used to determine the level of inflammation and the two methods showed a strong correlation (*r *= 0.807, n = 75, *P *< 0.001). LTβR-Ig-treated groups had reduced levels of inflammation as they aged and also the lowest inflammation levels compared with the controls at the different ages at which the mice were examined (Figure 1c, d). Collectively, the levels of inflammation increased as the NOD mice aged, with the oldest (19-week-old) untreated mice (NT19) recording the highest inflammation in the study (Figure 1c, d). In marked contrast to MOPC 21-treated and untreated mice, the inflammation levels (focus score and ratio index) in LTβR-Ig-treated mice never differed significantly from levels of healthy 9-week-old pre-disease mice. The inflammation levels of the LT19 group were significantly different from those of the NT19 group, but the comparison between LT19 and MO19 was not significant (Figure 1c, d). However, the main effect of treatment (comparing all LTβR-Ig-treated mice with all MOPC 21-treated groups using two-way between-groups ANOVA) was significant for both ratio index (*P *= 0.005) and focus score (*P *= 0.007).

After establishing that inflammation was indeed inhibited, we were interested in determining the specific components affected by the treatment and in establishing a time line for disease development. For these studies, an average of four mice per group were randomly selected to represent each treated group and each age. Tissue sections were prepared and appropriate antibodies were used to determine the specific cell phenotypes and structures (Tables 1 and 2).

**Table 1:** Absolute numbers of mice positive for high endothelial venules and follicular dendritic cell networks

	High endothelial venules			Follicular dendritic cells		
		
Treatment	LT	MO	NT	LT	MO	NT
Age in weeks						

9	-	-	0/4	-	-	0/4
12	1/4	1/4	2/4	0/4	0/4	2/4
16	0/4	4/4	3/4	0/4	1/4	3/4
19	0/4	3/4	2/3	0/4	2/4	1/3

Treatment with either lymphotoxin-beta receptor-immunoglobulin fusion protein (LTβR-Ig) or mouse monoclonal IgG1 (MOPC 21) was started at week 9. Euthanization was carried out 1 week after the last injection. Mice receiving either LTβR-Ig or MOPC 21 and euthanized at 12 weeks of age had received 3 injections, those euthanized at 16 weeks of age had received 7 injections, and those euthanized at 19 weeks of age had received 10 injections. -, no mice in these groups; LT, mice treated with lymphotoxin-beta receptor-immunoglobulin fusion protein; MO, mice treated with mouse monoclonal IgG1; NT, mice not treated.

**Table 2:** Absolute numbers of mice positive for chemokines

	CXCL13			CCL19					
		
Treatment	LT	MO	NT	LT		MO		NT	
				
Age in weeks				Acini/duct	Infiltrate	Acini/duct	Infiltrate	Acini/duct	Infiltrate
9	-	-	0/4	-	-	-	-	4/4	0/4
12	2/4	1/4	4/4	4/4	0/4	4/4	1/4	4/4	2/4
16	4/4	4/4	4/4	4/4	0/4	4/4	4/4	4/4	3/4
19	2/4	4/4	3/3	3/4	0/4	2/4	2/4	2/3	1/3

Treatment with either lymphotoxin-beta receptor-immunoglobulin fusion protein (LTβR-Ig) or mouse monoclonal IgG1 (MOPC 21) was started at week 9. Euthanization was carried out 1 week after the last injection. Mice receiving either LTβR-Ig or MOPC 21 and euthanized at 12 weeks of age had received 3 injections, those euthanized at 16 weeks of age had received 7 injections, and those euthanized at 19 weeks of age had received 10 injections. -, no mice in these groups; LT, mice treated with lymphotoxin-beta receptor-immunoglobulin fusion protein; MO, mice treated with mouse monoclonal IgG1; NT, mice not treated.

### LTβR-Ig treatment reduces the B- and T-cell zones in submandibular glands

**Figure 2:**
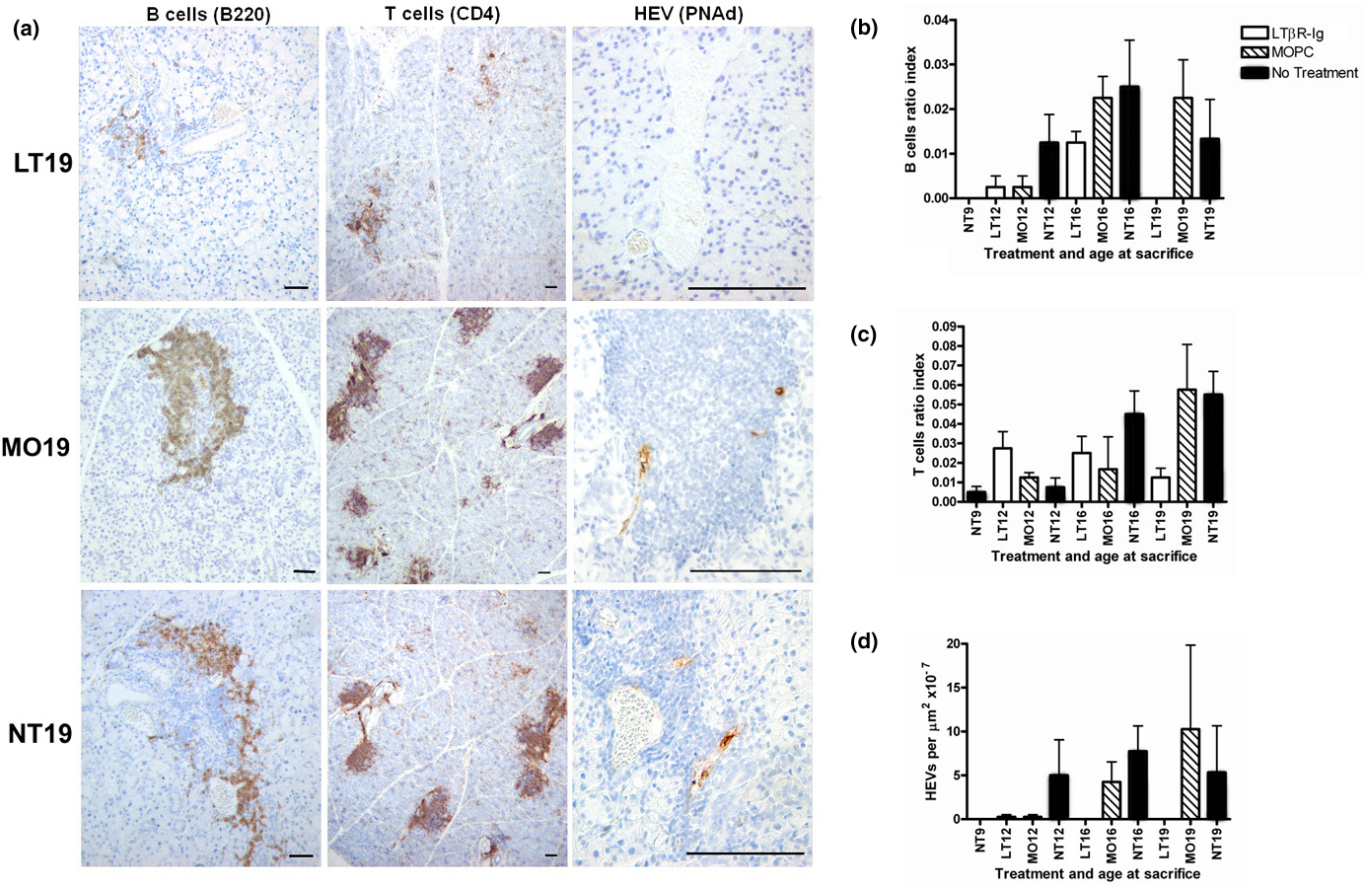
Effect of lymphotoxin-beta receptor-immunoglobulin fusion protein (LTβR-Ig) treatment on B-cell zones, T-cell zones, and high endothelial venules (HEVs). **(a) **Photomicrographs of immunohistochemical staining for B cells, T cells, and HEVs in the submandibular glands. Three or four mice from each of the 10 groups were randomly selected for immunohistochemistry. Data shown here are representative examples of control mice (19 weeks old) that were not treated (NT19) or that were treated with 10 injections of mouse monoclonal IgG1 (MOPC 21) (MO19) and of mice treated with 10 injections of LTβR-Ig (LT19). Bars = 100 μm. **(b) **Proportion of B-cell zones to the total area of the gland (that is, the B-cell ratio index). **(c) **Proportion of T-cell zones to the total area of the gland (that is, the T-cell ratio index). **(d) **Number of HEVs to the total area of the gland. Each of the 10 groups had three or four randomly selected mice for each staining and analysis. Legend applies to frames (b-d). Bars indicate the mean and the standard error of the mean.

There were no B-cell zones at the beginning of the experiment (NT9). These appeared in all of the groups at 12 weeks and peaked at 16 weeks of age (Figure 2a, b). At 19 weeks, the LTβR-Ig-treated group B-cell zones were almost completely inhibited (B-cell zones recorded in only one mouse in the LT19 group) (Figure 2b and data not shown). Statistical comparisons between MOPC 21 and LTβR-Ig groups indicate that B-cell zone ratios were influenced mainly by treatment type (*P *= 0.011) and number of injections (*P *= 0.037). Although the intergroup differences were not statistically significant, T-cell zones increased with age in the MOPC 21 and untreated groups but declined with age in the LTβR-Ig group (Figure 2a, c). The B-cell zones were smaller than T-cell zones (see y-axis of Figure 2a, b). When B-cell zones or T-cell zones were observed in LTβR-Ig-treated mice, these structures were composed of groups of closely packed cells concentrated at one pole of the infiltrate. Scattered T and B cells were also observed (Figure 2a).

### LTβR-Ig treatment inhibits high endothelial venules and follicular dendritic cell networks in submandibular glands

**Figure 3:**
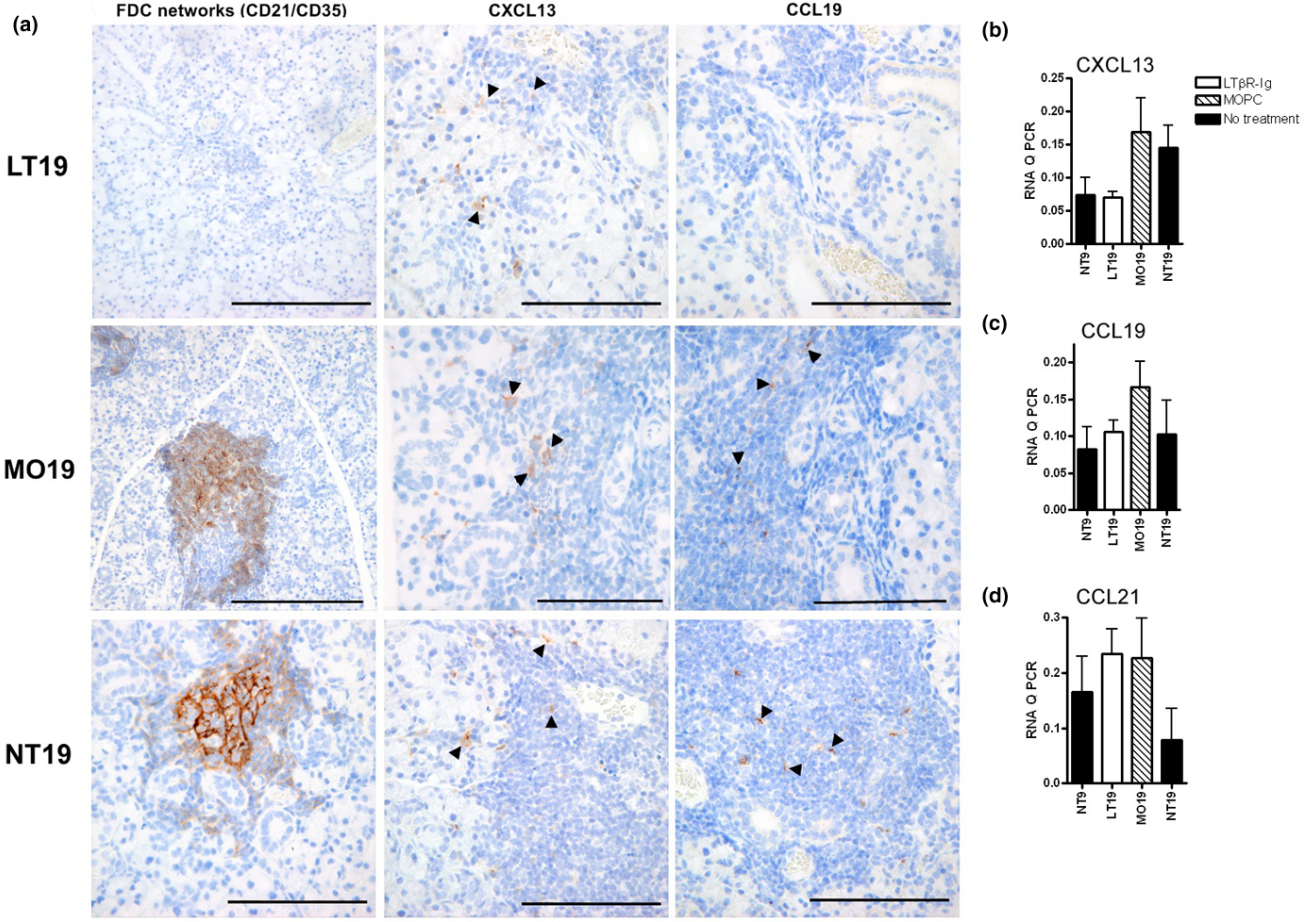
Effect of lymphotoxin-beta receptor-immunoglobulin fusion protein (LTβR-Ig) treatment on follicular dendritic cell (FDC) networks and chemokines. **(a) **Photomicrographs of immunohistochemical staining for FDC networks and CXCL13 and CCL19 in the submandibular glands. Three or four mice from each of the 10 groups were randomly selected for immunohistochemistry. Data shown here are representative examples of control mice (19 weeks old) that were not treated (NT19) or that were treated with 10 injections of mouse monoclonal IgG1 (MOPC 21) (MO19) and of mice treated with 10 injections of LTβR-Ig. Arrowheads indicate sites of chemokine staining. Bars = 100 μm. A comparison of relative mRNA expressions of CXCL13 **(b)**, CCL19 **(c)**, and CCL21 **(d) **in salivary glands of the baseline group (NT9 group) and the oldest mice (19 weeks old) treated with either LTβR-Ig or MOPC 21 or untreated. Salivary glands from seven or eight mice per group were used in this analysis. Quantitative polymerase chain reactions (Q PCRs) using RNA from each organ were run in quadruplicate, and the mean signal was normalized to the glyceraldehyde 3-phosphate dehydrogenase (GAPDH). Data are presented as the mean of the values for the replicate organs (seven or eight organs per point) and the standard deviation. The differences between LT19 and controls were not significant.

LTβR-Ig treatment inhibited/reversed development of HEVs (Table 1 and Figure 2a, d). In all of the LTβR-Ig-treated mice examined, only one HEV in one 12-week-old mouse was observed. FDC networks were first noted at 12 weeks of age in the untreated mice (NT12). Treatment with LTβR-Ig blocked development of FDC networks in the salivary glands examined (Table 1 and Figure 3a).

### The effect of LTβR-Ig treatment on chemokine mRNA expression and immunoreactive protein and the profound reduction of CCL19 protein

CXCL13 was present in the inflammatory infiltrates of all of the groups studied except the untreated 9-week-old mice (NT9) (Table 2 and Figure 3a). Some cells showed membranous staining but more commonly CXCL13 was noted as a dispersed substance within the infiltrates (Figure 3a). CCL19 was demonstrated in the salivary glands at sites of inflammation, more commonly as a dispersed substance within the infiltrate (Figure 3a). In some cells, CCL19 membranous staining was also observed (Figure 3a). CCL19 expression was also found on ductal and acinar epithelia (Table 2 and Figure 3a). Unlike CXCL13, CCL19 was not expressed in the infiltrate of any of the mice treated with LTβR-Ig (Table 2). The mRNA expression levels of CXCL13, CCL19, and CCL21 were also analyzed, as shown in Figure 3b–d. When we compared the chemokines in the LTβR-Ig-treated mice with those of age-matched controls or the baseline group (NT9), only CXCL13 was clearly downmodulated.

### Effects of LTβR-Ig treatment on lymph nodes

**Figure 4:**
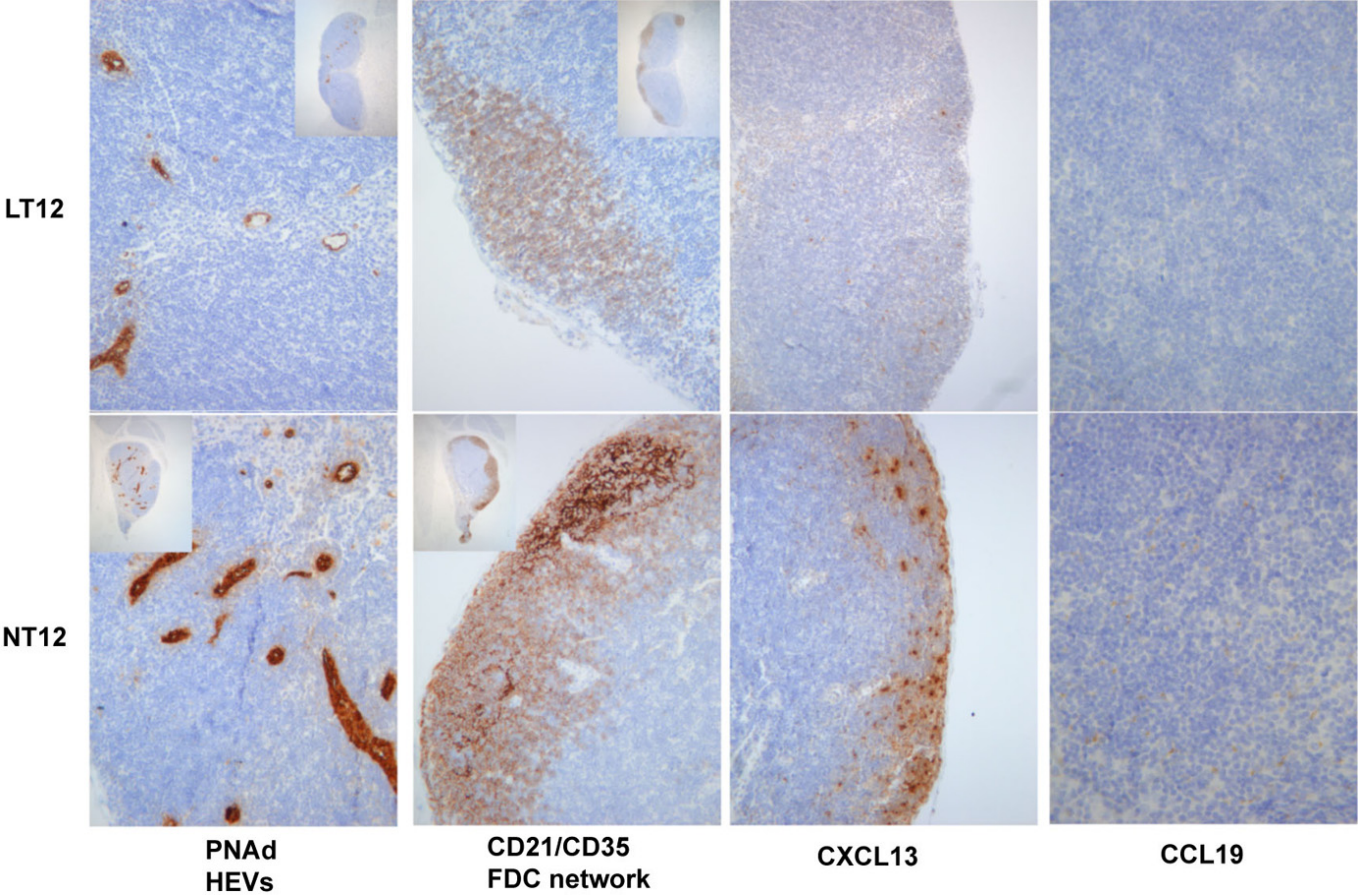
Immunohistochemical analysis of lymph nodes. Mandibular lymph nodes from two to six mice per group were examined (multiple lymph nodes in some mice). Representative photomicrographs of immunohistochemical staining of lymph nodes from mice from the LT12 group and NT12 group. For the follicular dendritic cell (FDC) network panels, the LT12 lymph node has no FDC networks, but B cells are picked by the CD21 antibody, and the NT12 lymph node has prominent FDC networks. Original magnifications: × 50 (insets), × 400 (CCL19), and × 200 (all other images).

In contrast to the salivary glands where no LTβR-Ig-treated mice had FDC networks, the mandibular lymph nodes were affected by LTβR-Ig treatment in a more gradual manner: Some disorganized or remnant networks were found in both LT12 (Figure 4) and LT16, but none was found in LT19 group (data not shown). The effect of LTβR-Ig treatment on CCL19 (Figure 4) was also less dramatic in the mandibular lymph nodes than in the salivary glands as this chemokine was expressed in some but not all lymph nodes in the LT16 and LT19 groups. Whereas HEVs in the salivary glands were very sensitive to LTβR-Ig treatment and were completely cleared in the LT16 and LT19 groups, HEVs as revealed by MECA-79 staining were diminished in the mandibular lymph nodes although some HEVs were still visible in the LT12 (Figure 4), LT16, and LT19 groups. These histological changes to the lymph nodes resemble those described previously following LTβR-Ig treatment [33]. CXCL13 was expressed in all of the draining lymph nodes from the LTβR-Ig-treated mice. However, more intense staining was noted in the controls than in the LTβR-Ig-treated mice (Figure 4), indicating the likelihood of a quantitative difference.

### Salivary gland function is partially restored after treatment with LTβR-Ig

**Figure 5:**
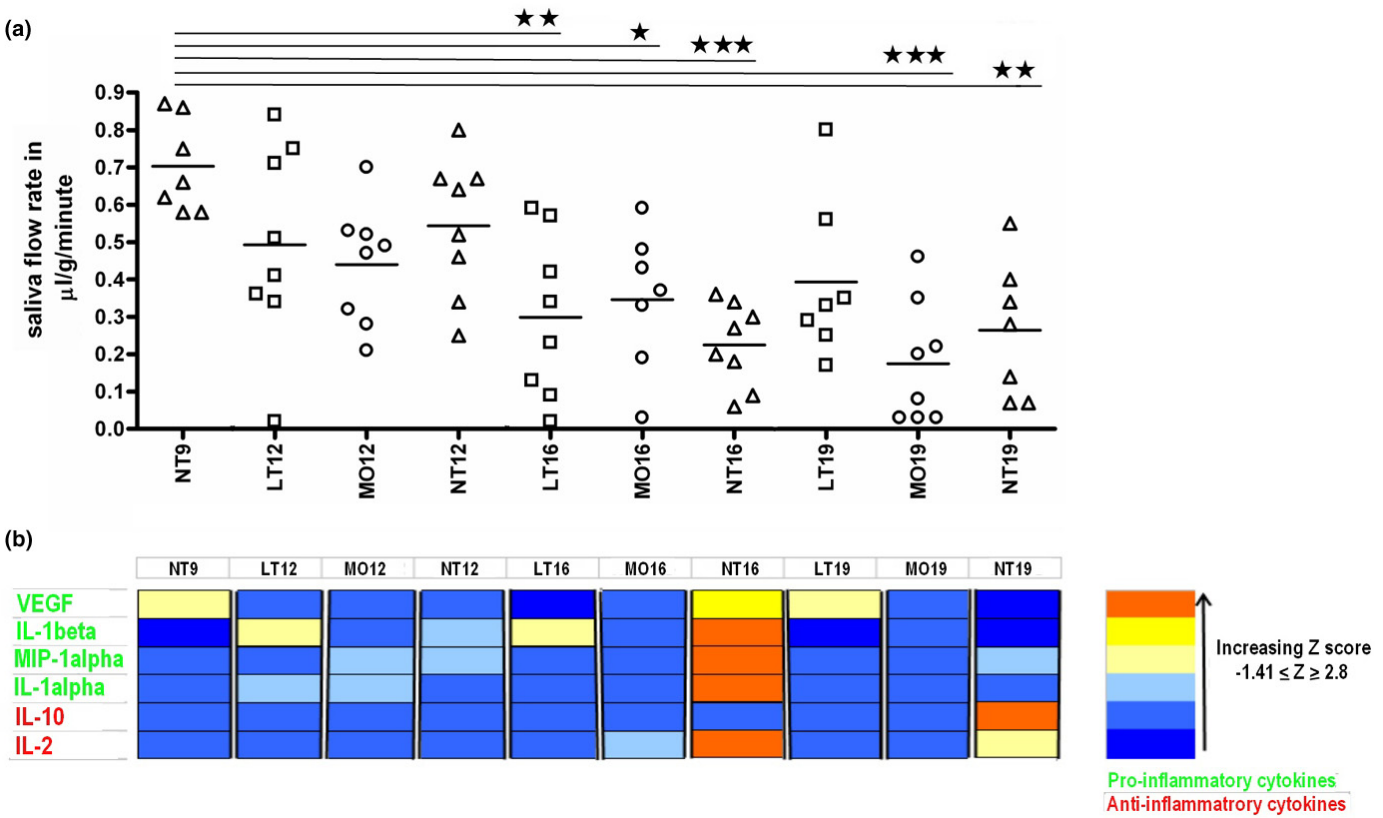
Salivary gland function and saliva cytokine analysis. **(a) **Saliva flow rates of the 10 groups. The saliva flow rate was expressed as microliters of saliva secreted per minute per gram of mouse body weight. Data were analyzed by one-way analysis of variance followed by Tukey *post hoc *test. *P < 0.05, **P < 0.01, and ***P < 0.001 indicate statistically significant differences between the specific group and the NT9 group. Horizontal lines indicate the mean. Symbols refer to individual mice treated with lymphotoxin-beta receptor-immunoglobulin fusion protein (LTβR-Ig) (□), mouse monoclonal IgG1 (MOPC 21) (○), or no treatment (Δ). Each symbol indicates data from one mouse. **(b) **Heat map of saliva cytokines showing fluctuations in the levels of cytokines in the control mice groups and LTβR-Ig-treated mice over time. The mean cytokine level of each mouse group was determined and Z-score standardization was computed for each cytokine using SPSS version 15.0. The heat map was drawn in Excel^® ^2003. IL, interleukin; MIP-1α, macrophage inflammatory protein-1 alpha; VEGF, vascular endothelial growth factor.

Generally, a subtle dichotomous distribution of saliva flow rates within each group was observed (Figure 5a). To determine whether saliva flow rate was restored, all of the groups of mice were compared with the pre-disease state (that is, untreated mice euthanized at 9 weeks [NT9]). Overall, the saliva flow rate declined in all of the groups of NOD mice that were 9 to 12 weeks old but this decline was not statistically significant (Figure 5a). At 16 weeks, all three groups had a significant decline in saliva flow (Figure 5a). At 19 weeks of age, the saliva flow rate of the LT19 group was partially restored to the levels recorded in the NT9 group (Figure 5a). This phenomenon did not occur in either the MO19 or the NT19 group. There was a statistically significant main effect on the saliva flow rate of the number of injections administered (*P *= 0.042). The saliva flow rates of the MO19 and LT19 groups were significantly different (*P *= 0.049). To determine whether the hyperglycemic state of the mice influenced the saliva flow rates, we compared the saliva flow rates in mice within the same group with either low or high glucose levels and found no significant differences (data not shown).

### Cytokines in saliva samples

Interleukin (IL)-1-beta was detected in all of the samples analyzed (from 55 individual mice), whereas vascular endothelial growth factor was detected in all but one of the samples. For macrophage inflammatory protein-1 alpha (MIP-1α), IL-10, IL-2, and IL-1α, samples with a mean fluorescent intensity corresponding to a value lower than the least detectable concentration were assigned values of 0. Basic fibroblast growth factor and CXCL10 were each detected in only one of the samples. The other cytokines/chemokines tested (that is, granulocyte macrophage-colony stimulating factor, interferon-gamma, IL-4, IL-5, IL-12, IL-13, IL-17, CXCL1, CCL2, CXCL9, and TNF-α) were not detected in any of the samples. There was a striking similarity between the saliva cytokine profile of the LT19 and NT9 groups. High levels of saliva cytokines (both pro- and anti-inflammatory) were observed in the NT16 group. The results of the analysis of the pro- and anti-inflammatory cytokines are shown in Figure 5b.

### Serum LTβR-Ig levels decrease as the mice age

The serum levels of LTβR-Ig were determined 1 week after the final LTβR-Ig injection in each group. A more-than-twofold drop in the LTβR-Ig levels was observed in older mice (88.19 ± 12.04 and 40.17 ± 8.87 μg/mL in 12-week-old and 19-week-old mice, respectively).

### Effect of mouse monoclonal IgG1

An effect of MOPC 21 was observed. However, except for expression levels of MO19 and NT19 CCL21b mRNA (*P *= 0.042), the levels of the various parameters studied in MOPC 21 mice were not significantly different from those of the untreated mice.

## Discussion

The results disclosed that blocking the LTβR pathway in NOD mice not only has an effect on the blood glucose levels, as reported previously [23,25], but also has profound effects on the salivary gland lesion, a hallmark of SS-like disease in NOD mice. The loss of FDC networks on blocking the LTβR pathway in adult mice has been described previously [19,34], but the present study is the first to report that LTβR-Ig treatment results in FDC network loss in the salivary glands. FDC networks constitutively release a substantial amount of CXCL13 [35], although there are other sources of CXCL13, such as macrophages, dendritic cells, and primed follicular helper T cells [35]. CXCL13 recruits B cells and follicular helper T cells to the B-cell zones. Immunohistochemistry disclosed that both LTβR-Ig-treated mice and controls expressed CXCL13 at the protein level in the salivary glands. There was, however, a depletion of B-cell zones by 19 weeks (LT19 group). This was probably attributable to the reduction in the amount of CXCL13, as indicated by the reduction in CXCL13 mRNA expression levels in the LT19 group compared with controls. The complete loss of FDC networks following LTβR-Ig treatment may also have contributed to the lack of B-cell zones in the LT19 group.

There was also an all-out loss of HEVs in the salivary glands in the LT16 and LT19 groups. HEVs exhibit specific address signals that are recognized by circulating lymphocytes. PNAd is one such signal (antigen) that was first defined by reactivity with monoclonal antibody MECA-79 [36]. L-selectin on the circulating lymphocytes binds carbohydrate epitopes of PNAd, facilitating homing of these naïve lymphocytes [37]. Previous studies have reported a prominent role for the LTβR pathway in maintaining the HEV structures in a functional state in lymph nodes of adult mice [13,33] and in sites of lymphoid neogenesis [38]. The LTβR pathway is also important in *de novo *lymphangiogenesis in sites of lymphoid neogenesis [39].

Although the mRNA expression levels of CCL19 were not significantly reduced in the LTβR-Ig-treated mice, there was a complete loss of CCL19 protein in the salivary gland infiltrates of LTβR-Ig-treated mice. CCL19 and CCL21 are necessary for the transmigration of naïve T cells and central memory T cells across the HEVs and in the formation of T-cell zones [40]. Therefore, the combination of ablation of FDC networks, loss of HEVs, and reduced chemokine expression would ultimately result in a reduced influx of B and T cells to the salivary glands. This may explain the reduced B- and T-cell zones in the LTβR-Ig-treated mice.

Another interesting observation was the different response to LTβR-Ig treatment by the salivary gland lesion and the draining lymph nodes. There is no consensus as to which of these sites drives the immune response or whether there is a contribution from both [13]. Our findings indicate that the LTβR-Ig treatment had a more profound effect on the microarchitecture of the salivary gland lymphoid tissue than on that of the lymph node.

Our findings also suggest that the salivary gland lesion has a prominent role in SS-like disease, as partial saliva flow restoration occurs in association with the phenotypic changes in the salivary gland. The ectopic lymphoid tissue has a prominent role in several diseases/conditions [13]. In the NOD mouse, pancreatic lymphoid neogenesis could initiate and sustain the progression of IDDM in the absence of draining lymph nodes [41]. Interestingly, it has been proposed that LTβR-Ig treatment would result in a quiescent state of the lymph nodes, draining the sites of inflammation [13]. This is partly supported by the present study, showing microarchitectural differences between LTβR-Ig-treated mice and control lymph nodes. However, the lymph node microarchitectural changes in our experimental setup are unlike the grossly disturbed primary or secondary organ development observed in mice deficient in components of the LTβR pathway.

The present study was based on the hypothesis that LTβR-Ig treatment would preserve the integrity of the salivary gland by preventing development and progression of inflammation and lymphoid neogenesis, thus preventing the occurrence of salivary gland dysfunction. Our results, however, show that LTβR-Ig treatment may reverse pathological events that have already occurred in the salivary gland. This possibility is underscored by the finding of HEVs in the salivary glands of 12-week-old mice given three LTβR-Ig injections (although in only one mouse) but loss of HEVs in 16-week-old and 19-week-old mice treated with LTβR-Ig. Earlier analyses in mice suggested that a period of at least 2 weeks of LTβR-Ig treatment was required to downmodulate HEVs and therefore any early HEVs could still be present at week 12 [33]. Also, the saliva flow rate appeared to fall (comparing LT12 and LT16 groups) but recovered in the LT19 group, indicating that the salivary gland dysfunction may have been prevented or reversed.

The dichotomous distribution of saliva flow rates in NOD mice has been described previously [42]. The same study also used saliva proteins to describe the efficacy of intervention agents in SS-like disease of NOD mice. In our study, the striking similarity in the cytokine profiles of the pre-disease NT9 group and the LT19 group may indicate a restoration of the pre-disease quality of saliva in the LT19 group. However, analysis of pro- and anti-inflammatory cytokines and chemokines in saliva from untreated mice across different ages disclosed no obvious trend indicative of progressive disease status. However, 16 weeks may mark a time of potentially heightened cytokine production and activity in the NOD mouse.

Accelerated clearance of immunoglobulin-based agents is commonly observed in many rodent autoimmune disease models, and our finding of a reduction in the serum levels of LTβR-Ig as the mice age also points to a possible increased clearance of the fusion protein in these older NOD mice. It is postulated that, had the high levels of LTβR-Ig observed in 12-week-old mice been sustained throughout the experiment, the protective/reversal effects of LTβR-Ig would have been more pronounced.

There are severe technical limitations to finding proper controls for fusion proteins when faced with the need to use high-level dosing in autoimmune disease models to maintain coverage. A protective effect of polyclonal human IgG used as a control protein in experiments to determine the efficacy of LTβR-Ig in collagen-induced arthritis was reported [34]. The mechanism behind the MOPC 21 effect may involve non-specific binding of MOPC 21 to targets in the pathways involved in the disease process, resulting in a protective effect.

The immune dysregulation occurring in the female NOD background leads to autoreactivity and inflammation of the salivary glands. This inflammation is accompanied by the formation of lymphoid tissues in the salivary glands with HEVs and FDC networks. Although the role of these tertiary lymphoid tissues in the pathogenesis remains unclear, the present study clearly demonstrates that LTβR inhibition, whether via its effects on trafficking or lymphoid architecture, results in a less destructive environment in the target organ.

## Conclusion

This study shows that blocking the LTβR pathway in NOD mice ablates lymphoid neogenesis in the salivary glands and this is accompanied by an improvement in salivary gland function. To our knowledge, the effect of interfering with the LTβR axis has not previously been explored in SS-like disease. It is hoped that our results will contribute to the development of appropriate methods for treatment of human SS.

## Abbreviations

ANOVA: analysis of variance; BSA: bovine serum albumin; CCL: C-C chemokine ligand; CXCL: C-X-C chemokine ligand; FDC: follicular dendritic cell; HEV: high endothelial venule; IDDM: insulin-dependent diabetes mellitus; IL: interleukin; LIGHT: a cytokine homologous to lymphotoxins that shows inducible expression and competes with herpes simplex virus glycoprotein D for herpes virus mediator (HVEM), a receptor induced on T cells; LT: lymphotoxin; LTβR: lymphotoxin-beta receptor; LTβR-Ig: lymphotoxin-beta receptor-immunoglobulin fusion protein; MOPC 21: mouse monoclonal IgG1; NOD: non-obese diabetic; NT: not treated; PBS: phosphate-buffered saline; PNAd: peripheral (lymph) node addressin; QPCR: quantitative polymerase chain reaction; SS: Sjögren syndrome; TNF: tumor necrosis factor.

## Competing interests

JLB and AP are employees of Biogen Idec. The other authors declare that they have no competing interests.

## Authors' contributions

AIB designed the study and helped to carry out the animal experiments, to analyze the results, and to write the paper. MKG helped to carry out the animal experiments, performed the immunohistochemistry, enzyme-linked immunosorbent assay, multiplex analysis, and statistics, and helped to perform the morphometric analysis, to analyze the results, and to write the paper. KS helped to carry out the animal experiments, to perform the morphometric analysis, to analyze the results, and to write the paper. AP performed the QPCR and helped to analyze the results. JLB and RAF helped to analyze the results and to write the paper. All authors read and approved the final manuscript.

## References

[B1] Fox RI (2005). Sjögren's syndrome. Lancet.

[B2] Stott DI, Hiepe F, Hummel M, Steinhauser G, Berek C (1998). Antigen-driven clonal proliferation of B cells within the target tissue of an autoimmune disease. The salivary glands of patients with Sjögren's syndrome. J Clin Invest.

[B3] Salomonsson S, Jonsson MV, Skarstein K, Brokstad KA, Hjelmström P, Wahren-Herlenius M, Jonsson R (2003). Cellular basis of ectopic germinal center formation and autoantibody production in the target organ of patients with Sjögren's syndrome. Arthritis Rheum.

[B4] Jonsson MV, Szodoray P, Jellestad S, Jonsson R, Skarstein K (2005). Association between circulating levels of the novel TNF family members APRIL and BAFF and lymphoid organization in primary Sjögren's syndrome. J Clin Immunol.

[B5] Jonsson MV, Skarstein K, Jonsson R, Brun JG (2007). Serological implications of germinal center-like structures in primary Sjögren's syndrome. J Rheumatol.

[B6] Browning JL, Androlewicz MJ, Ware CF (1991). Lymphotoxin and an associated 33-kDa glycoprotein are expressed on the surface of an activated human T cell hybridoma. J Immunol.

[B7] Browning JL, Dougas I, Ngam-ek A, Bourdon PR, Ehrenfels BN, Miatkowski K, Zafari M, Yampaglia AM, Lawton P, Meier W (1995). Characterization of surface lymphotoxin forms. Use of specific monoclonal antibodies and soluble receptors. J Immunol.

[B8] Crowe PD, VanArsdale TL, Walter BN, Ware CF, Hession C, Ehrenfels B, Browning JL, Din WS, Goodwin RG, Smith CA (1994). A lymphotoxin-beta-specific receptor. Science.

[B9] Mauri DN, Ebner R, Montgomery RI, Kochel KD, Cheung TC, Yu G-L, Ruben S, Murphy M, Eisenberg RJ, Cohen GH, Spear PG, Ware CF (1998). LIGHT, a new member of the TNF superfamily, and lymphotoxin alpha are ligands for herpesvirus entry mediator. Immunity.

[B10] Ware CF (2008). Targeting lymphocyte activation through the lymphotoxin and LIGHT pathways. Immunol Rev.

[B11] De Togni P, Goellner J, Ruddle NH, Streeter PR, Fick A, Mariathasan S, Smith SC, Carlson R, Shornick LP, Strauss-Schoenberger J (1994). Abnormal development of peripheral lymphoid organs in mice deficient in lymphotoxin. Science.

[B12] Ettinger R, Browning JL, Michie SA, van Ewijk W, McDevitt HO (1996). Disrupted splenic architecture, but normal lymph node development in mice expressing a soluble lymphotoxin-beta receptor-IgG1 fusion protein. Proc Natl Acad Sci USA.

[B13] Browning JL (2008). Inhibition of the lymphotoxin pathway as a therapy for autoimmune disease. Immunol Rev.

[B14] Koni PA, Sacca R, Lawton P, Browning JL, Ruddle NH, Flavell RA (1997). Distinct roles in lymphoid organogenesis for lymphotoxins alpha and beta revealed in lymphotoxin beta-deficient mice. Immunity.

[B15] Alimzhanov MB, Kuprash DV, Kosco-Vilbois MH, Luz A, Turetskaya RL, Tarakhovsky A, Rajewsky K, Nedospasov SA, Pfeffer K (1997). Abnormal development of secondary lymphoid tissues in lymphotoxin beta-deficient mice. Proc Natl Acad Sci USA.

[B16] Futterer A, Mink K, Luz A, Kosco-Vilbois MH, Pfeffer K (1998). The lymphotoxin beta receptor controls organogenesis and affinity maturation in peripheral lymphoid tissues. Immunity.

[B17] Rennert PD, Browning JL, Mebius R, Mackay F, Hochman PS (1996). Surface lymphotoxin alpha/beta complex is required for the development of peripheral lymphoid organs. J Exp Med.

[B18] Scheu S, Alferink J, Potzel T, Barchet W, Kalinke U, Pfeffer K (2002). Targeted disruption of LIGHT causes defects in costimulatory T cell activation and reveals cooperation with lymphotoxin beta in mesenteric lymph node genesis. J Exp Med.

[B19] Gommerman JL, Browning JL (2003). Lymphotoxin/LIGHT, lymphoid microenvironments and autoimmune disease. Nat Rev Immunol.

[B20] Kratz A, Campos-Neto A, Hanson MS, Ruddle NH (1996). Chronic inflammation caused by lymphotoxin is lymphoid neogenesis. J Exp Med.

[B21] Aloisi F, Pujol-Borrell R (2006). Lymphoid neogenesis in chronic inflammatory diseases. Nat Rev Immunol.

[B22] Drayton DL, Liao S, Mounzer RH, Ruddle NH (2006). Lymphoid organ development: from ontogeny to neogenesis. Nat Immunol.

[B23] Wu Q, Salomon B, Chen M, Wang Y, Hoffman LM, Bluestone JA, Fu Y-X (2001). Reversal of spontaneous autoimmune insulitis in nonobese diabetic mice by soluble lymphotoxin receptor. J Exp Med.

[B24] Levisetti MG, Suri A, Frederick K, Unanue ER (2004). Absence of lymph nodes in NOD mice treated with lymphotoxin-{beta} receptor immunoglobulin protects from diabetes. Diabetes.

[B25] Ettinger R, Munson SH, Chao C-C, Vadeboncoeur M, Toma J, McDevitt HO (2001). A critical role for lymphotoxin-beta receptor in the development of diabetes in nonobese diabetic mice. J Exp Med.

[B26] Hu Y, Nakagawa Y, Purushotham KR, Humphreys-Beher MG (1992). Functional changes in salivary glands of autoimmune disease-prone NOD mice. Am J Physiol.

[B27] Cha S, Nagashima H, Brown VB, Peck AB, Humphreys-Beher MG (2002). Two NOD Idd-associated intervals contribute synergistically to the development of autoimmune exocrinopathy (Sjögren's syndrome) on a healthy murine background. Arthritis Rheum.

[B28] Johansson AC, Nakken B, Sundler M, Lindqvist AK, Johannesson M, Alarcón-Riquelme M, Bolstad AI, Humphreys-Beher MG, Jonsson R, Skarstein K, Holmdahl R (2002). The genetic control of sialadenitis versus arthritis in a NOD.Q × B10.Q F2 cross. Eur J Immunol.

[B29] Hjelmervik T, Lindqvist A-K, Petersen K, Johannesson M, Stavrum A-K, Johansson A, Jonsson R, Holmdahl R, Bolstad A (2007). The influence of the NOD Nss1/Idd5 loci on sialadenitis and gene expression in salivary glands of congenic mice. Arthritis Res Ther.

[B30] Gommerman JL, Giza K, Perper S, Sizing I, Ngam-ek A, Nickerson-Nutter C, Browning JL (2003). A role for surface lymphotoxin in experimental autoimmune encephalomyelitis independent of LIGHT. J Clin Invest.

[B31] Jonsson R, Tarkowski A, Bäckman K, Holmdahl R, Klareskog L (1987). Sialadenitis in the MRL-l mouse: morphological and immunohistochemical characterization of resident and infiltrating cells. Immunology.

[B32] Skarstein K, Johannessen AC, Holmdahl R, Jonsson R (1997). Effects on sialadenitis after cellular transfer in autoimmune MRL/lpr mice. Clin Immunol Immunopathol.

[B33] Browning JL, Allaire N, Ngam-ek A, Notidis E, Hunt J, Perrin S, Fava RA (2005). Lymphotoxin-beta receptor signaling is required for the homeostatic control of HEV differentiation and function. Immunity.

[B34] Fava RA, Notidis E, Hunt J, Szanya V, Ratcliffe N, Ngam-ek A, de Fougerolles AR, Sprague A, Browning JL (2003). A role for the lymphotoxin/LIGHT axis in the pathogenesis of murine collagen-induced arthritis. J Immunol.

[B35] Allen CD, Cyster JG (2008). Follicular dendritic cell networks of primary follicles and germinal centers: phenotype and function. Semin Immunol.

[B36] Streeter PR, Rouse BT, Butcher EC (1988). Immunohistologic and functional characterization of a vascular addressin involved in lymphocyte homing into peripheral lymph nodes. J Cell Biol.

[B37] Berg EL, Robinson MK, Warnock RA, Butcher EC (1991). The human peripheral lymph node vascular addressin is a ligand for LECAM-1, the peripheral lymph node homing receptor. J Cell Biol.

[B38] Luther SA, Lopez T, Bai W, Hanahan D, Cyster JG (2000). BLC expression in pancreatic islets causes B cell recruitment and lymphotoxin-dependent lymphoid neogenesis. Immunity.

[B39] Furtado GC, Marinkovic T, Martin AP, Garin A, Hoch B, Hubner W, Chen BK, Genden E, Skobe M, Lira SA (2007). Lymphotoxin beta receptor signaling is required for inflammatory lymphangiogenesis in the thyroid. Proc Natl Acad Sci USA.

[B40] Ebert LM, Schaerli P, Moser B (2005). Chemokine-mediated control of T cell traffic in lymphoid and peripheral tissues. Mol Immunol.

[B41] Lee Y, Chin RK, Christiansen P, Sun Y, Tumanov AV, Wang J, Chervonsky AV, Fu Y-X (2006). Recruitment and activation of naive T cells in the islets by lymphotoxin [beta] receptor-dependent tertiary lymphoid structure. Immunity.

[B42] Delaleu N, Madureira AC, Immervoll H, Jonsson R (2008). Inhibition of experimental Sjögren's syndrome through immunization with HSP60 and its peptide amino acids 437–460. Arthritis Rheum.

